# A case report of advanced small intestinal stromal tumor with KIT gene mutation and BRCA2 deletion after multi-line treatments

**DOI:** 10.3389/fonc.2025.1630699

**Published:** 2025-08-01

**Authors:** Shiyun Cui, Lei Fan, Yu Bai, Xinnan Sun, Yucheng Cai, Jingqi Dai, Ting Wang, Chongqi Sun, Rong Wang, Lianke Liu

**Affiliations:** ^1^ Department of Oncology, The First Affiliated Hospital of Nanjing Medical University, Jiangsu, Nanjing, China; ^2^ Department of Oncology, Chongqing Hospital of Jiangsu Province Hospital, The People’s Hospital of Qijiang District, Chongqing, China; ^3^ Department of General Surgery, Affiliated Hospital of Integrated Traditional Chinese and Western Medicine, Nanjing University of Chinese Medicine, Jiangsu, Nanjing, China; ^4^ Department of General Surgery, Jiangsu Province Academy of Traditional Chinese Medicine, Jiangsu, Nanjing, China; ^5^ Department of Clinical Medicine, Nanjing Medical University, Jiangsu, Nanjing, China

**Keywords:** gastrointestinal stromal tumor, gene mutation, targeted therapy, multidisciplinary treatment, intestinal stromal tumor

## Abstract

This study reports a 40-year-old male with small intestinal stromal tumor (SIST). After initial surgery and adjuvant imatinib, the tumor recurred. The patient then received multiple rounds of treatment with targeted drugs and surgical interventions. Through comprehensive analysis of gene mutation profiles (KIT and HRR gene mutations, including BRCA2), a combination therapy of fluzoparib, pamiparib, and ripretinib was administered, stabilizing the patient’s condition with significant efficacy. This case highlights the importance of genetic testing and personalized targeted treatment strategies for gastrointestinal stromal tumor (GIST) patients.

## Introduction

Gastrointestinal stromal tumor (GIST) is a rare mesenchymal tumor whose pathogenesis highly depends on activating mutations in tyrosine kinase genes. 80% of GISTs harbor KIT or PDGFRA mutations driving tumorigenesis (4–6). Diagnosis of GIST relies primarily on histopathology and immunohistochemistry, with CD117 and CD34 positivity serving as key diagnostic criteria (7). Standard treatments include surgical resection for localized disease and tyrosine kinase inhibitors (imatinib/sunitinib/regorafenib) for advanced cases (8–12). Although targeted therapy has expanded the treatment options for GIST and improved the efficacy, the problems of tumor recurrence and drug resistance still seriously hinder the long-term survival and prognosis improvement of patients. The GIST patient in this case has a complex condition with multiple relapses and genetic testing results including common KIT gene mutation and rare BRCA2 deletion. By deeply analyzing this case, this paper explores the importance and implementation path of genetic testing and individualized targeted treatment strategies in the disease management of GIST, aiming to provide references for clinical treatment.

## Case report

### Initial diagnosis and treatment

A 40-year-old male with no significant medical/family history presented with abdominal discomfort in June 2015. Physical examination showed no obvious abnormalities. He underwent small intestine tumor resection and end-to-side anastomosis, revealing an 8×6×6 cm exophytic jejunal tumor (2 cm from Treitz ligament) with capsule rupture and focal bleeding. The physical examination was normal after the operation and postoperative pathology confirmed high-risk GIST (13×6.5×5 cm, mitotic count >5/50 HPF, reactive mesenteric lymph nodes, CD117 (+); pT4N0M0, stage IIIB) with no co-morbidities. He received first-line imatinib (400mg/day) per guidelines (1), continuing for 3 years before discontinuation.

### First recurrence

In November 2019, the patient’s regular reexamination by CT showed a pelvic mass, which was considered a recurrence of stromal tumor. Therefore, the patient resumed imatinib treatment (400mg/day) in hopes of controlling tumor progression.

### Disease progression and second-line treatment

In August 2020, despite continuous imatinib treatment, the tumor still showed signs of progression. To remove the lesions as much as possible, the patient underwent pelvic tumor resection, small intestine mesentery tumor resection, and greater omentum tumor resection. After the operation, imatinib maintenance treatment (400mg/day) was continued.

### Re-recurrence and subsequent treatments

In November 2022, the tumor progressed again, and sunitinib was administered as second-line therapy (2). A CT scan on December 1, 2022, revealed left mid-lower abdominal retroperitoneal and intra-abdominal masses consistent with recurrent/metastatic stromal tumor with bleeding, showing increased size, abdominopelvic effusion, hemoperitoneum, and exudative changes. The patient underwent mesenteric artery embolization, splenic angiography, and abdominal tumor radioactive particle implantation for local control, followed by third-line regorafenib (3). Despite these interventions, the tumor continued to progress.

### Gene testing-guided precision medicine

**Figure 1:**
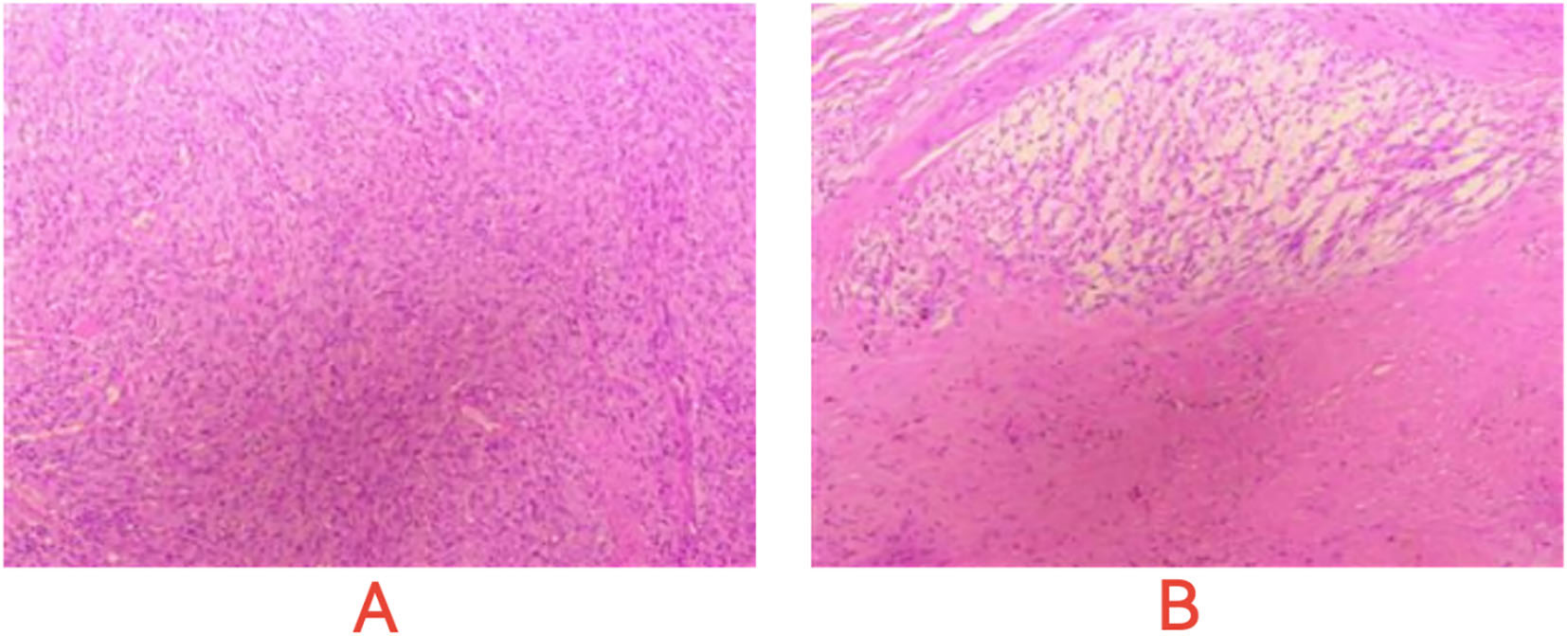
Microscopic images of post - operative pathological sections. **(A)** Dense, interlaced spindle cells with uniform nuclei, suggesting GIST’s mesenchymal traits. **(B)** Cell clusters and necrosis-like areas, indicating GIST heterogeneity and evolution signs.

On January 13, 2023, a CT scan revealed left abdominal irregular masses with mixed density, heterogeneous enhancement, and intestinal wall thickening, confirming disease progression. Tissue Sample & Detection Methodology: FFPE tumor tissue from small intestinal resection (January 2023) underwent genomic profiling with strict quality control (H&E-confirmed ≥100 viable tumor cells, >20% tumor content). Molecular characterization employed: NGS: OncoD-C1021T panel (Illumina NovaSeq 6000, 500× depth, VAF sensitivity ≥0.5%) IHC: PD-L1 22C3 pharmDx assay (Dako Autostainer Link 48) with dual scoring (TPS/CPS).A comprehensive genetic test identified KIT exons 11/13/17 mutations (p.V559D:33.8%, p.V654A:27.5%, p.Y823D:2.1%) and BRCA2 deletion (copy number coefficient: 0.7) with additional HRR gene alterations (RAD54L, FANCM, RAD51B, RAD51 deletions; TMB-L, MSS). The patient started combination therapy with fluzoparib and ripretinib, achieving significant abdominal pain relief within 2 weeks. However, at day 55, lower gastrointestinal bleeding occurred (colonoscopy: colonic fistula). CT showed partial lesion shrinkage, enlargements with liquefactive necrosis. Emergency surgery (left hemicolectomy, retroperitoneal resection, colostomy) confirmed GIST on pathology (spindle cell tumors with bleeding/necrosis. **Figure 1**). Immunohistochemistry showed:(Abdominal cavity stromal tumor + left half colon) Tumor cells CD117(+), CD34(-), DOG-1(-), S-100(-), SMA(+), Ki67 (approximately 20%),SDHA(partial+), SDHB(+);(Tumor nodule anterior to left kidney) Tumor cells CD117(+), CD34(-), DOG-1(-), S-100(-), SMA(+), Ki67(approximately10%), SDHA(partial+), SDHB(+).

Postoperative genetic retesting in April 2023 confirmed persistent KIT exon 11/13/17 mutations and BRCA2 deletion (**Table 1**), with no newly acquired mutations. The treatment plan was adjusted to continue fluzoparib-ripretinib combination in April 2023 for further tumor control.

**Table 1:** The genetic testing results.

Detection Results for Efficacy Prediction of PARP Inhibitors	
Indicator/Gene	Testing Results
BRCA1	–
BRCA2	Deficient (0.7)
ATM	–
ATR	–
ATRX	–
BAP1	–
BARD1	–
BLM	Deficient (0.7)
BRIP1	–
CDK12	–
CHEK2	Deficient (0.7)
C11orf30	–
BRCC1	–
FAM175A	–
FANCA	–
FANCC	–
FANCD2	–
FANCE	–
FANCF	–
FANCG	–
FANCL	–
FANCM	Deficient (0.6)
MRE11A	–
NBN	–
PALB2	–
RAD50	–
RAD51	Deficient (0.7)
RAD51B	Deficient (0.7)
RAD51C	–
RAD51D	–
RAD52	–
RAD54L	Deficient (0.7)
RECQL	–
RECQL4	–
WRN	–
BRAF	–
EGFR	–
ERBB4	–
TP53	–
PTEN	–

The dash “-” indicates that no variations related to targeted drug use were found in this detection.

### Subsequent treatment adjustment

**Figure 2:**
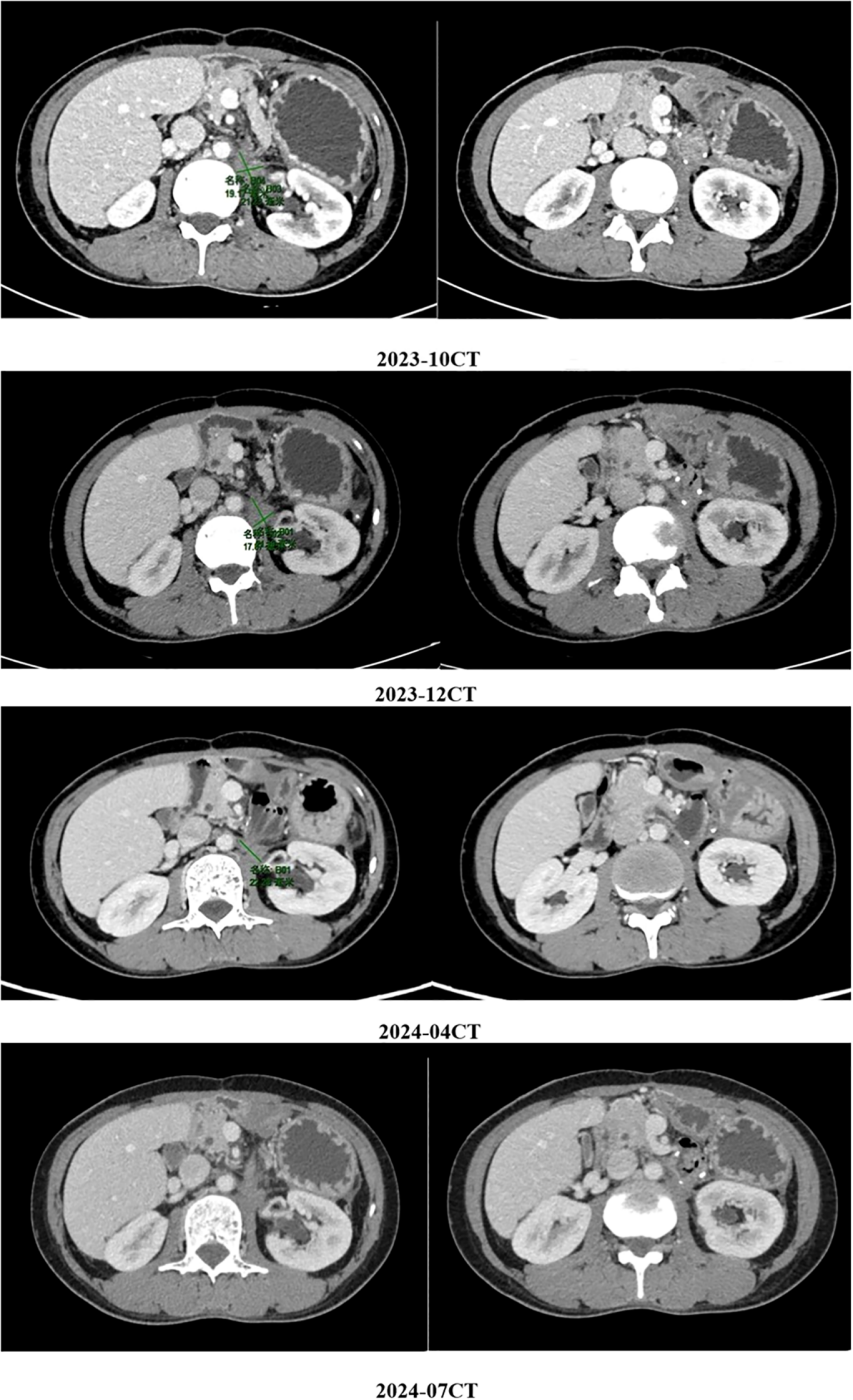
The patient’s condition remained stable, and there were no obvious signs of disease progression.

**Figure 3:**
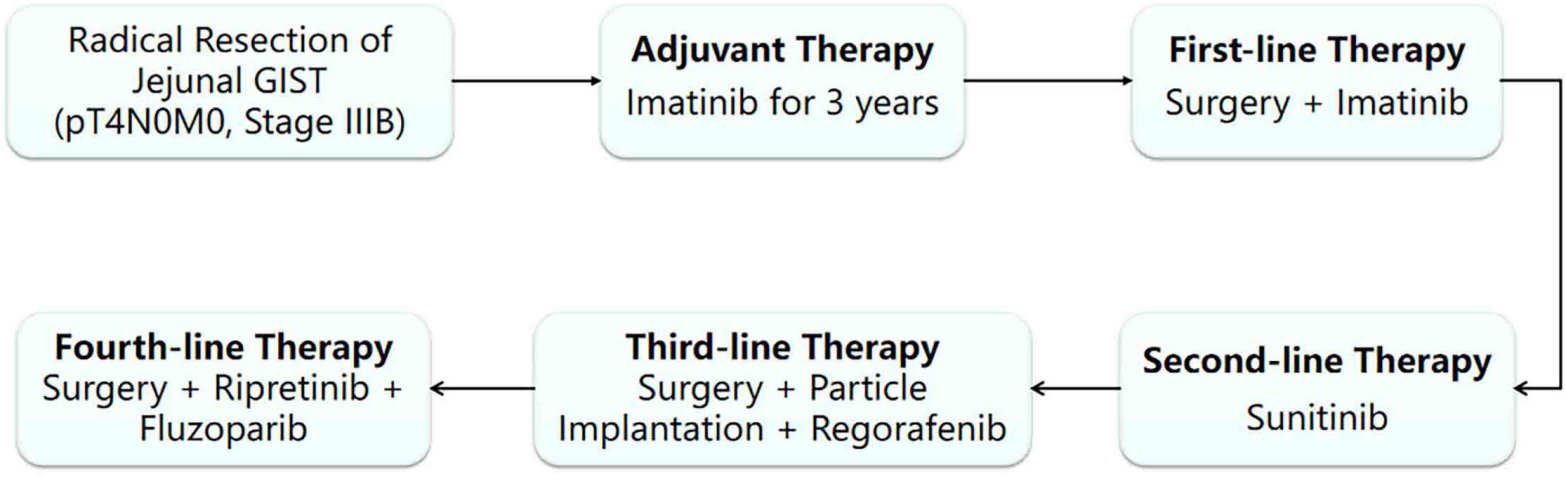
Treatment flowchart.

The above combined treatment continued until June 2023. The treatment was suspended because the patient’s hemoglobin dropped to 75 g/L. The treatment resumed in July 2023. On August 29th 2023, a CT reexamination showed new soft tissue density shadows around the anastomosis, with no significant enhancement post-contrast, suggesting necrotic or cystic components rather than active tumor proliferation. The combined treatment of fluzoparib and ripretinib was continued. During the regular reexamination by CT, the disease was stable. Considering the adverse reactions during the previous treatment process and the patient’s overall tolerance, in September 2023, the medical team adjusted the treatment plan to a combined treatment of pamiparib and ripretinib. During the subsequent regular follow-up, the patient’s condition remained stable, and there were no obvious signs of disease progression (**Figures 2**, **3**).

## Discussion

GIST is the most common mesenchymal tumor with multidirectional differentiation in the digestive tract, mainly occurring in the stomach and small intestine with nonspecific symptoms (4). The annual incidence is approximately 1–2 cases per 100,000 population, peaking at 50–60 years of age with no significant gender predilection. Originating from gastrointestinal pacemaker cells, it is linked to c-kit and PDGFRα mutations, with potential malignancy (5, 6). GIST has a potential malignant transformation tendency, and its risk assessment depends on tumor size, location, and mitotic count. Diagnosis relies on histopathology and immunohistochemistry (7). Treatment includes surgery and targeted agents (8, 9): imatinib as first-line inhibits KIT/PDGFRα (10), sunitinib as second-line targets VEGFR/PDGFR (11), and regorafenib as third-line blocks multiple tumor kinases (12).

Drug resistance to targeted agents is a core challenge in advanced GIST, as exemplified by this case. Tumor recurrence after initial imatinib suggests incomplete eradication of minimal residual disease or secondary KIT mutations. Short-lived efficacy and resistance to re-challenged imatinib, plus failure of sunitinib/regorafenib, correlate with dynamic KIT mutation evolution. KIT exon 17 mutations alter imatinib binding sites, reducing sensitivity (13). While sunitinib/regorafenib may target some secondary mutations (14), the patient’s mutation profile led to treatment failure. BRCA2 deletion accelerates resistant clone evolution via genomic instability, exacerbating therapeutic challenges.

The key turning point of this case is that genetic testing revealed mutations in exons 11/13/17 of the KIT gene and HRR gene mutations including the deletion of BRCA2 (LOH), providing important targets for subsequent treatment. BRCA1/2 gene mutations have been extensively studied in malignant tumors such as breast cancer and ovarian cancer, but are extremely rare in GIST. In 2015, a case report described a patient with BRCA2 gene mutation who had prostate cancer, breast cancer, and GIST simultaneously, thus proposing a possible association between GIST and BRCA2 (15). In 2017, a case report described an extremely rare case of familial GIST with germline KIT mutations coexisting with hereditary breast and ovarian cancer syndrome (HBOC). The simultaneous presence of two distinct germline mutations gave rise to different familial neoplastic diseases, yet the report did not characterize the association between them (16). In this current case, the patient has both KIT gene mutations and homologous recombination deficiency (HRD) - related changes such as BRCA2 deletion. BRCA2 copy number 0.7 suggests biallelic inactivation, correlating directly with PARP inhibitor sensitivity (NCCN guidelines). Compound HRD (RAD54L deletion 0.6 + CHEK2 deletion 0.7) exacerbates genomic instability, potentially accelerating clonal expansion of low-frequency mutations such as KIT Y823D (2.1%). The genetic characteristics are more complex and have been rarely reported in the existing literature. According to international GIST genomics research, approximately 80% of GISTs are caused by driver mutations in KIT or PDGFRA. The remaining cases may be associated with SDH deficiencies or other rare gene variations, and HRD - related BRCA mutations have not yet been included in the routine genetic testing scope or molecular typing system for GIST (17). This phenomenon indicates that HRD - related BRCA mutations may not be the driving events of GIST, but rather accompanying genetic alterations, and their clinical significance remains to be further explored.

The BRCA2 deletion in this case represents a somatic, acquired alteration rather than a germline pathogenic/likely pathogenic variant. This conclusion is supported by: (i) absence of personal/family history indicating hereditary cancer syndromes; (ii) lack of BRCA2 abnormalities in the initial 2015 tumor specimen, with deletion first detected after multi-line TKI therapies; and (iii) co-deletion of multiple HRR genes (RAD54L, FANCM, RAD51B, RAD51), suggesting genomic instability-driven somatic evolution. The acquisition of BRCA2 loss may be driven by TKI treatment pressure [promoting clonal selection of repair-deficient subpopulations, analogous to secondary KIT mutations (13, 14)], BRCA2 haploinsufficiency (accelerating mutagenesis to facilitate resistance), and potentially therapeutic mutagenesis from prior interventions, though direct evidence is limited. This dynamic genomic evolution underscores the necessity for repeated genetic profiling in advanced GIST to identify acquired therapeutic vulnerabilities.

As a key HRR pathway gene, BRCA2 mutation causes homologous recombination repair defects, making tumor cells dependent on PARP-mediated single-strand repair. PARP inhibitors exert ‘synthetic lethality’ to kill such cells. BRCA mutations may accelerate KIT secondary mutations in GIST, promoting imatinib resistance, while PARP inhibitors target these repair-deficient cells. PARP inhibitors have proven effective in BRCA-mutated ovarian and breast cancers (18, 19), with consistent mechanisms in GIST. This case studies showed fluzoparib rapidly relieves symptoms in BRCA-mutated GIST, offering a new strategy for TKI-resistant patients. Meanwhile, further exploration of immunotherapy can be carried out. HRR gene mutations may increase the tumor mutation burden (TMB) and enhance the efficacy of immune checkpoint inhibitors (20). In the future, combined treatment with PARP inhibitors and immunotherapy can be attempted. Notably, dynamic genetic monitoring is essential. Multiline-resistant GIST patients should undergo expanded genetic testing (including HRR-related genes like BRCA) to identify therapeutic targets.

When evaluating treatment efficacy, it is crucial to balance toxic and side effects management. In this case, fluzoparib showed efficacy but caused lower gastrointestinal bleeding. As a PARP inhibitor, it suppresses tumor cell DNA repair and promotes apoptosis, yet may also disrupt DNA repair in normal cells, compromising gastrointestinal mucosal and vascular integrity. Ripretinib’s broad-spectrum inhibition of KIT mutations may further aggravate gastrointestinal toxicity. Timely surgical intervention and treatment adjustment (suspending fluzoparib and switching to pamiparib combined with ripretinib) mitigated risks while controlling the tumor.

The case’s success stemmed from integrating surgical debulking, interventional therapies (embolization, particle implantation), and precise targeted treatments. Surgical resection during local tumor progression relieved symptoms and provided fresh samples for genetic testing, ensuring result reliability. This dynamic, genetic testing - and multidisciplinary - collaboration - based decision - making model should supplement the advanced GIST standardized treatment system.

Future research could focus on: 1) investigating HRR mutation prevalence in GIST through large - scale sequencing and its prognostic significance; 2) using in - vitro experiments to determine if HRD - associated BRCA deficiency impacts KIT mutations or signaling pathways; 3) optimizing PARP inhibitor application in HRR - mutated GIST, including timing, dosage, combinations, and adverse reaction prevention.

## Conclusions

The rarity of mutations in HRR genes such as BRCA in GIST highlights the uniqueness of this case. The discovery of this mutation provides crucial clues for individualized treatment. Although the existing evidence is limited, this genetic feature may become a new target for breaking through the drug resistance bottleneck. In the future, it is necessary to further clarify the biological role of HRR genes mutations in GIST through multi-center cooperation and basic research, and optimize the precision treatment strategies for such patients.

## Data Availability

The original contributions presented in the study are included in the article/supplementary material. Further inquiries can be directed to the corresponding author.
